# Decoding psychotropic-induced metabolic disturbances: gut-brain axis, multi-omics, and nutrition-lifestyle integration

**DOI:** 10.3389/fnut.2026.1778794

**Published:** 2026-03-31

**Authors:** Dhoha Dhieb, Kholoud Bastaki

**Affiliations:** Pharmaceutical Sciences Department, College of Pharmacy, QU Health, Qatar University, Doha, Qatar

**Keywords:** gut microbiota, gut-brain-metabolic axis, insulin resistance, metabolic disturbances, multi-omics, nutrition, precision psychiatry, psychotropic medications

## Abstract

Psychotropic medications remain central to psychiatric treatment, yet their use is frequently accompanied by a substantial metabolic burden. Metabolic adverse effects, including weight gain, insulin resistance, dysglycemia, dyslipidemia, and hypertension, complicated clinical management and undermine long-term adherence. Although evidence-based monitoring and mitigation approaches exist, the molecular determinants of individual susceptibility and the drivers of interindividual variability in metabolic outcomes remain insufficiently defined. Emerging evidence identifies the gut-brain-metabolic axis as a key mechanistic interface, with psychotropic medications altering gut microbiota and associated metabolic pathways that contribute to metabolic complications. Multi-omics strategies are beginning to illuminate the complex molecular networks underlying these adverse effects; however, most findings still arise from isolated omics layers, limiting mechanistic resolution and translational utility. Integrative analytical frameworks, including artificial intelligence, now enable the synthesis of molecular, clinical, environmental, lifestyle, and dietary factors to support more precise and individualized intervention. In this narrative review, we synthesize current clinical and mechanistic advances in understanding psychotropic-induced metabolic dysfunction, with a focus on insulin resistance and the gut–brain–metabolic axis and highlight how multi-omics, environmental factors and computational strategies may advance future precision approaches in psychiatric care.

## Graphical abstract

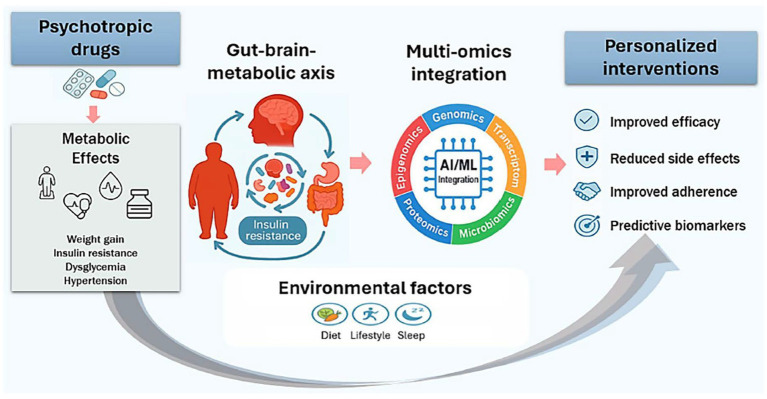

## 1 Introduction

Psychotropic medications are a cornerstone in the management of severe psychiatric disorders, including schizophrenia, bipolar disorder, and major depressive disorder. Their clinical benefits are undeniable; however, these agents frequently impose a substantial metabolic burden, manifesting as weight gain, insulin resistance (IR), dysglycemia, dyslipidemia, and hypertension. These complications significantly increase long-term morbidity and mortality, while also reducing quality of life and treatment adherence (1–3). Weight gain has been considered the primary metabolic concern, yet emerging evidence demonstrates that metabolic disturbances can occur independently of adiposity, suggesting direct pharmacologic effects on systemic metabolic regulation (4, 5). Second-generation antipsychotics (SGAs) are consistently associated with the highest metabolic risk among psychotropic agents. These effects are not limited to appetite dysregulation but reflect a broader and more complex pathophysiology. Current research implicates multiple biological systems, including mitochondrial function, inflammatory and immune signaling, and peripheral tissue metabolism, as key contributors to psychotropic-induced metabolic dysfunction. These pleiotropic effects can impair cellular energy homeostasis, promote chronic low-grade inflammation, and disrupt hormonal and metabolic signaling, ultimately driving IR and cardiometabolic complications even in the absence of significant weight gain (1, 6, 7). Emerging evidence also highlights the gut-brain-metabolic axis as an additional layer of complexity, with psychotropic medications altering gut microbiota composition and intestinal barrier integrity, thereby accelerating systemic inflammation and metabolic dysregulation (5, 8). It is important also to note that individuals with psychiatric disorders often exhibit elevated baseline metabolic vulnerability due to disease biology, lifestyle factors, and chronic inflammation, amplifying susceptibility to medication-induced metabolic injury (4, 9). This multifactorial risk landscape underscores the inadequacy of monitoring strategies that rely solely on weight change.

Although expanding literature applies multi-omics profiling, spanning genomics, epigenomics, transcriptomics, proteomics, metabolomics and metagenomics, to psychotropic-associated metabolic adverse effects, most findings remain compartmentalized within individual data layers and heterogeneous study designs, constraining mechanistic integration and clinical translation (10, 11). Integrative computational approaches, including artificial intelligence, offer a route to combine molecular signatures with clinical trajectories and environmental and lifestyle exposures for risk stratification and early detection; however, robust external validation, attention to confounding and bias, and prospective evaluation are required before routine clinical implementation (12, 13). In this narrative review, we synthesize current clinical and mechanistic insights into psychotropic-induced metabolic disturbances, with a focus on IR and the emerging role of the gut-brain-metabolic axis. We further discuss how multi-omics, environmental factors, and artificial intelligence may inform precision strategies to manage and prevent these metabolic complications in psychiatric populations.

## 2 Psychotropic medication-induced metabolomic disturbances and insulin resistance

### 2.1 Clinical Spectrum and drug-specific liability

Individuals with mental illness, including schizophrenia, bipolar disorder, and major depressive disorder, experience markedly increased premature mortality, with life expectancy shortened by up to 20 years and mortality rates approximately 2.0–3.5 times that of the general population (5, 14). A key modifiable contributor to this excess burden is the emergence of psychotropic medication–associated metabolic dysregulation, presenting as coordinated disturbances in body weight and fat distribution, glycaemic control (IR, hyperglycaemia, and a trajectory toward prediabetes/diabetes), lipid handling (hypertriglyceridaemia, broader dyslipidaemia), and blood pressure regulation. These disturbances frequently manifest as metabolic syndrome but may also appear as earlier or incomplete phenotypes (3, 4, 15–20). Baseline vulnerability is considerable; for example, in the United States, the prevalence of obesity is approximately 28% among those with major depressive disorder or bipolar disorder, and up to 50% among individuals with schizophrenia (21). Notably, metabolic risk is present even prior to pharmacological treatment in SMI populations (5), suggesting that medication exposure exacerbates rather than initiates underlying metabolic vulnerability. Metabolic risk is heterogenous across psychotropic agents. Antidepressant-induced weight gain is generally less pronounced than with antipsychotics but is clinically significant for several specific agents, notably tricyclic antidepressants (e.g., amitriptyline, nortriptyline), certain Selective Serotonin Reuptake Inhibitors SSRIs (paroxetine, citalopram, fluvoxamine), mirtazapine, and the Monoamine Oxidase Inhibitors (MAOI) phenelzine (3, 15, 17). Mood stabilizers, particularly valproate, meaningfully contribute to cumulative risk, with weight gain detected in up to 70% of treated adults (15), and further associations with IR, type 2 diabetes, dyslipidaemia, and hypertension (4, 15, 21).

In contrast, antipsychotics, especially SGAs, are distinguished by their high metabolic liability. Up to 80% of SMI patients treated with SGAs experience significant weight gain, often within weeks of initiation (5, 15, 17). Liability is highly molecule-specific: clozapine and olanzapine confer the greatest risk for weight gain, hyperglycaemia, and dyslipidaemia, while quetiapine ranks intermediately, and other SGAs (e.g., ziprasidone, lurasidone, aripiprazole, cariprazine, brexpiprazole) are somewhat lower risk, but not metabolically neutral (2, 4, 5). Comparative syntheses reinforce this heterogeneity. Systematic review and network meta-analysis of 402 randomized controlled trials in schizophrenia, Huhn et al. reported that 12 of 26 antipsychotics (46%) caused significantly greater weight gain than placebo, with marked between-drug differences, zotepine produced the largest mean gain (3.21 kg, 95% CI 2.10–4.31), whereas haloperidol was among the lowest (0.54 kg, 95% CI 0.15–0.95); olanzapine and sertindole also ranked among agents associated with greater weight gain relative to most comparators (22). Longitudinal data converge on an early “high-risk” window after initiation. Pérez-Iglesias et al. followed first-treated psychotic episodes randomized to olanzapine, haloperidol or risperidone for up to 3 years and identified the first year as the most critical period for weight gain and metabolic abnormalities (23). De Hert et al. similarly reported that most weight gain occurs within the first few months in drug-naïve schizophrenia after antipsychotic initiation (24). Focusing on olanzapine specifically, Millen et al. analyzed 86 clinical trials and found the greatest weight gain during the first 3 months, continuing thereafter at a reduced rate, with a plateau at ~6–12 months (25). Collectively, these studies suggest that metabolomic trajectories are often established early and then stabilize, emphasizing prevention at initiation rather than later remediation. Metabolic syndrome offers a clinically standardized lens on this liability. Its prevalence among antipsychotic-treated populations ranges from 37 to 63% (19), with IR central to the clustering of hyperglycemia and dyslipidemia (18). Importantly, dysregulated blood pressure is now recognized as a core metabolic phenotype, not merely a cardiovascular sequel, emphasizing the multisystem nature of psychotropic-induced metabolic disturbance (4). These findings underpin consensus recommendations for systematic metabolic monitoring in all patients undergoing antipsychotic therapy (26, 27).

### 2.2 Mechanistic basis of medication-induced metabolic dysfunction

Psychotropic medication–associated metabolic disturbances arise from convergent, multi-system mechanisms that extend beyond caloric intake and weight gain alone. Across drug classes, most prominently with SGAs such as clozapine and olanzapine, evidence supports pleiotropic effects on mitochondrial bioenergetics, oxidative and inflammatory stress pathways, neuroendocrine and appetite-regulatory receptor signaling, and peripheral tissue metabolic programs, with additional contributions from gut microbiome remodeling (6, 28–33). These processes interact across organs (liver, adipose tissue, skeletal muscle, and pancreas) and can collectively promote insulin resistance, dyslipidaemia, ectopic lipid deposition, and downstream cardiometabolic risk.

#### 2.2.1 Mitochondrial dysfunction and oxidative stress

A growing body of evidence implicates dose-dependent mitochondrial impairment as a central mechanism. SGAs (especially clozapine and olanzapine) have been shown to decrease oxygen consumption, accelerate ATP depletion, and elevate reactive oxygen species (ROS) in key metabolic organs, such as the liver, adipose tissue, and skeletal muscle (1, 6, 31, 32). These effects translate into cellular energy deficits and the downstream production of ROS, which can damage mitochondrial membranes and propagate lipid peroxidation and mitochondrial DNA (mtDNA) damage (34, 35). Notably, clozapine and olanzapine demonstrate the most pronounced mitochondrial toxicity, with quetiapine showing intermediate effects and risperidone and aripiprazole exerting milder impacts (1). This is clinically relevant, as heightened mitochondrial toxicity is associated with cumulative metabolic risk and, in certain instances such as quetiapine overdose, marked increases in serum myeloperoxidase (MPO) and C-reactive protein (CRP), correlating with clinical severity (32). Importantly, mitochondrial dysfunction does not operate in isolation; chronic low-grade inflammation both potentiates mitochondrial injury and is reciprocally amplified by ROS production. Dysfunctional mitochondria can act as sources of damage-associated molecular patterns (DAMPs) that activate cytokine production (IL-6, TNF-α), contributing to a self-reinforcing cycle of metabolic injury (6, 35, 36). While these processes are most marked with high-risk agents, SSRIs, typically considered low risk, have also been linked to moderate mitochondrial dysfunction and increased oxidative stress during long-term administration (37, 38).

#### 2.2.2 Inflammation and immune dysregulation

Low-grade systemic inflammation is now recognized as both a driver and consequence of psychiatric medication–induced metabolic dysfunction, with SGAs exerting the largest effects (1, 6, 36). Elevated pro-inflammatory cytokines and acute-phase reactants (CRP, IL-6, TNF-α) further impair mitochondrial function, and their persistent elevation has been linked to cardiovascular and metabolic complications. Mechanistically, these effects often parallel the receptor binding affinities of SGAs, both at the hypothalamic level and within target peripheral tissues, including adipose and hepatic tissue (30). Quetiapine provides a notable example of context-dependent inflammatory modulation; it can suppress inflammation in CNS microglia but enhance adipose cytokine production, underscoring the complexity of tissue-specific effects (39). Furthermore, long-term SSRI use has been associated with persistent, albeit milder, inflammatory signatures and may contribute to antidepressant treatment resistance (31, 38, 40). In treatment-resistant depression (TRD), inflammatory activation may be relevant beyond metabolic risk because it can also reduce antidepressant responsiveness via a few convergent pathways. Pro-inflammatory cytokines (e.g., TNF-α, IL-6, IL-1β) can lower monoaminergic tone by increasing monoamine transporter activity, while inflammation-induced indoleamine 2,3-dioxygenase (IDO) shifts tryptophan metabolism toward the kynurenine pathway, promoting oxidative/glutamatergic stress and reduced neurotrophic support (41–50). Chronic inflammation may additionally impair catecholamine synthesis through depletion of tetrahydrobiopterin (BH4) and disrupt neurotransmitter handling, collectively contributing to reduced brain-derived neurotrophic factor (BDNF), dependent plasticity and poor treatment response in inflammatory phenotypes (51–55). We note TRD briefly to emphasize inflammation as a shared mechanistic hub linking psychotropic response variability with metabolic vulnerability.

#### 2.2.3 Neurotransmitter receptor pathways and appetite regulation

Medication effects on appetite and energy balance remain important, particularly for SGAs. Antagonism at histamine H1 and serotonin 5-HT2C receptors disrupts hypothalamic satiety signaling, increases caloric intake, and reduces energy expenditure, contributing to weight gain and downstream metabolic deterioration (2, 30). Clozapine and olanzapine are the highest-affinity antagonists at these receptors, mirroring their clinical risk profiles for weight gain and dysmetabolism. By contrast, quetiapine and risperidone, with intermediate and lower affinities, respectively, confer proportionately reduced weight gain, while aripiprazole, which minimally interact with these sites, exhibits the most favorable metabolic profile among SGAs (2). SSRIs, while sparing H1 and 5-HT2C antagonism, can influence metabolic parameters via other mechanisms, including modulation of peripheral mitochondrial calcium flux and oxidative phosphorylation, as well as chronic effects on adipokine and insulin signaling (38). Acute SSRI use can transiently enhance insulin release, but chronic exposure has been associated with increased risk of IR and weight gain (8, 38).

#### 2.2.4 Peripheral tissue and organ system involvement

Beyond central mechanisms, peripheral tissues, particularly the liver, adipose tissue, and skeletal muscle, are key targets of psychiatric medication-induced metabolic toxicity. In the liver, SGAs impair mitochondrial complexes and reduce PPARα-regulated fatty acid β-oxidation, promoting lipid accumulation, non-alcoholic fatty liver disease (NAFLD), and dyslipidaemia (1, 8, 35). This aligns with the established role of PPARα as a master regulator of hepatic mitochondrial and peroxisomal β-oxidation, fatty acid uptake, and triglyceride catabolism (56, 57). Crosstalk between PPARα and lipogenic transcription factors such as SREBP-1c may further potentiate hepatic triglyceride storage. In adipose tissue, PPARγ is a central regulator of adipogenesis and adipokine secretion (57). Dysregulation of this pathway by high-liability SGAs (notably olanzapine and clozapine) promotes adipocyte hypertrophy, recruitment of pro-inflammatory macrophages, and impaired insulin signaling, mechanisms that collectively amplify systemic insulin resistance (6, 36, 39). Consistent with the literature, PPARγ influences insulin sensitivity through regulation of TNF-α, GLUT4 expression, and adipokines including adiponectin and resistin, pathways known to modulate glucose uptake and inflammatory tone (56, 58–60). In skeletal muscle, SGAs may interfere with PPARδ and its coactivator PGC-1α, diminishing oxidative mitochondrial capacity, reducing substrate oxidation, and impairing glucose homeostasis (1, 35). This mechanism compounds the reduction in insulin-stimulated glucose uptake associated with high-risk antipsychotics. Notably, activation of PPARδ normally enhances fatty acid oxidation and metabolic flexibility, functions reported in the PPAR literature to be crucial for maintaining insulin responsiveness (57). Importantly, PPAR signaling does not act in isolation but intersects with inflammatory cascades (e.g., TNF-α, IL-6), nutrient-sensing pathways such as AMPK, and gut-derived lipid mediators. This integration positions the PPAR family as a mechanistic bridge between psychotropic exposure and coordinated dysfunction across liver, adipose tissue, and skeletal muscle, ultimately leading to insulin resistance, dyslipidaemia, and ectopic fat deposition (6, 36, 39). Evidence from metabolic studies showing that PPARα and PPARγ agonists (e.g., fenofibrate, rosiglitazone) improve glucose, insulin, and triglyceride levels in diabetic animal models further underscores their central role in systemic metabolic regulation (61).

#### 2.2.5 Gut microbiome

Recent evidence increasingly implicates the gut microbiome as an important contributor to psychotropic medication-associated metabolic disturbances, acting in concert with mitochondrial dysfunction, inflammatory signaling, and peripheral tissue metabolic impairment (28). Preclinical and clinical studies show that high-liability second-generation antipsychotics, particularly olanzapine and clozapine, are associated with consistent alterations in gut microbial composition, including reduced short-chain fatty acid-producing taxa and an increased Firmicutes/Bacteroidetes ratio, changes linked to impaired glucose and lipid metabolism (28). In patients with schizophrenia, these microbial shifts are associated with elevated circulating lipopolysaccharide levels, insulin resistance, and hepatic steatosis, supporting the relevance of gut-derived signals to systemic metabolic risk (33, 62). By contrast, evidence for other antipsychotics such as quetiapine, risperidone, and aripiprazole remains limited, although available data suggest drug-specific microbial patterns accompanied by low-grade inflammatory responses (28). Selective serotonin reuptake inhibitors have also been shown to influence gut microbial composition beyond effects on gastrointestinal motility, with potential metabolic relevance (38). The broader implications of these microbiome alterations, including their integration with epigenomic, metabolomic, and nutrition-lifestyle factors, as well as their potential translational relevance, are examined in greater depth in sections (1, 2, 6, 28, 32–36, 38, 39, 62).

## 3 Microbiome and the gut-brain-metabolic axis in psychotropic induced metabolomic disturbance

A growing body of work links SGAs to gut microbiome perturbations that are implicated in antipsychotic-induced metabolomic disturbances, particularly weight gain and changes in glucose and lipid handling (63–66). In both rodent and human studies, SGA administration frequently results in shifts in community composition, with the most consistent signature being an increased Firmicutes:Bacteroidetes (F:B) ratio (67–70). This mirrors early observations in the obesity literature (71) where elevated F:B ratio is thought to facilitate increased energy harvest and fat storage (72, 73). However, findings at the phylum and taxa levels are inconsistent across studies in pediatric (74) and adult (75) populations reflecting the influence of cofounding factors such as age, diet, baseline community structure (76–78). As a result, there is growing recognition that microbial functional capacity, assessed through metabolic pathways, gene content and host–microbe signaling metabolites, may offer greater mechanistic insight than taxonomy alone. Within the gut–brain–metabolic axis, gut microbiota interact with the host via multiple endocrine, neural, and immune mechanisms. Microbial metabolites such as short-chain fatty acid (SCFAs) and indole derivatives, can act locally on enteroendocrine and immune cells and, after absorption, modulating appetite, hepatic glucose production, peripheral insulin sensitivity and systemic inflammation (79). Microbial regulation of bile acid metabolism and maintenance of intestinal barrier integrity create additional routes by which drug-induced dysbiosis may precipitate systemic metabolic disturbances, manifesting as IR, dyslipidemia, or ectopic fat deposition, beyond the effects of increased food intake alone.

**Figure 1:**
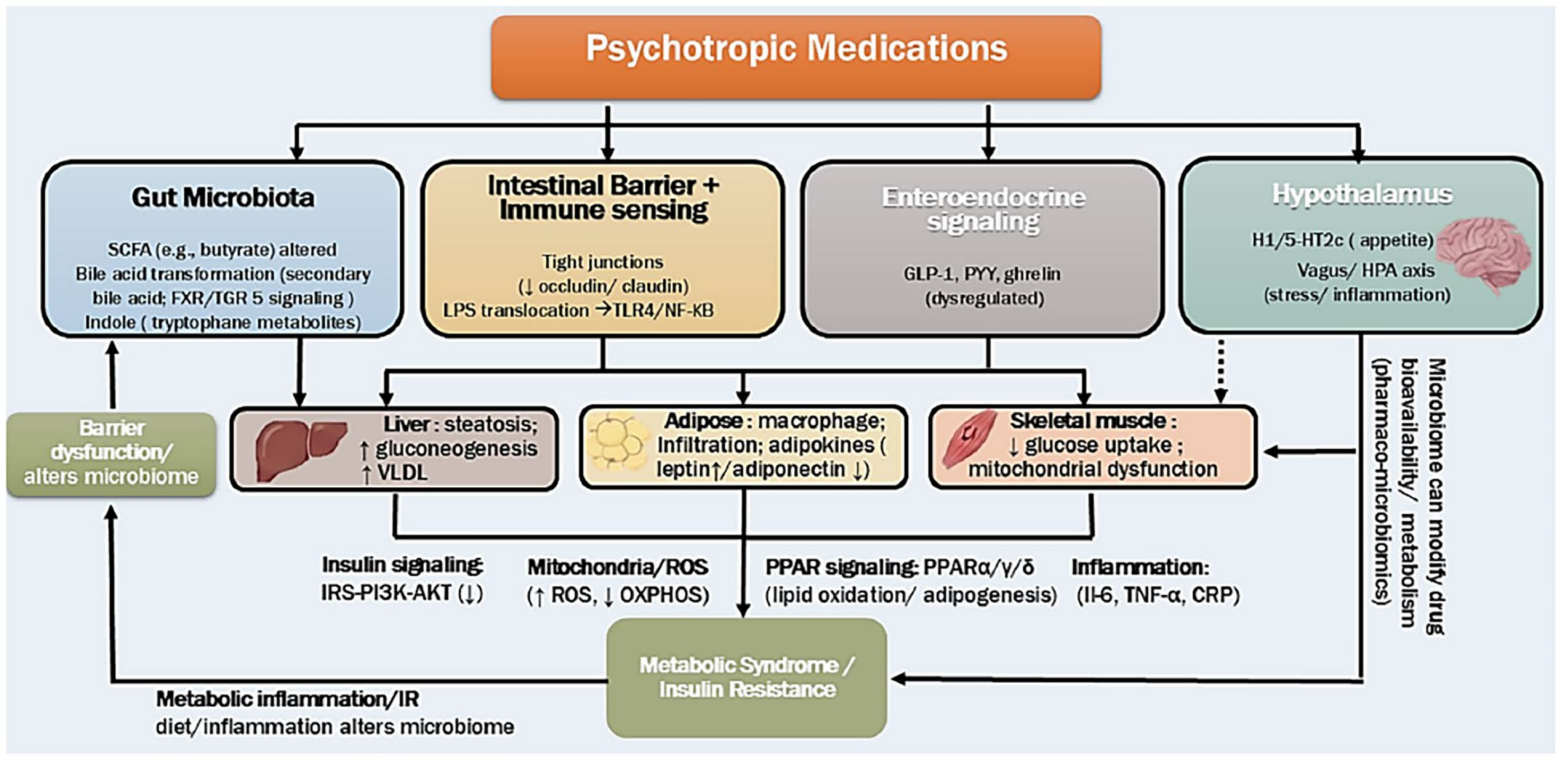
Mechanistic pathways linking psychotropic medications, gut microbiota, the gut–brain axis, and metabolic dysfunction. Psychotropic medications can remodel gut microbial functions and impair intestinal barrier integrity, increasing LPS translocation and innate immune activation (TLR4/NF-κB). These signals interact with enteroendocrine and hypothalamic regulation and converge on liver, adipose tissue, and skeletal muscle to promote inflammation, mitochondrial/oxidative stress, and impaired insulin signaling (IRS–PI3K–AKT), culminating in insulin resistance/metabolic syndrome. Bidirectional arrows indicate feedback to the gut ecosystem and potential microbiome–drug interactions affecting bioavailability/metabolism.

Preclinical experiments robustly support the causal role of microbiome alterations in antipsychotic-induced metabolic side effects. In germ-free mice, olanzapine-associated weight gain is absent but can be induced following colonization with gut microbes (68). Antibiotic administration can attenuate SGA-induced weight gain, adipose inflammation, and alter lipid metabolism, providing additional evidence for a causal role of the microbiome (69, 70). Similar results have been reported with risperidone, where microbiota changes contribute to reduced energy expenditure and the phenotype is transferable by fecal transplantation (63). In clinical population, Bahr et al. demonstrated that risperidone treatment in children and adolescents led to an increased F:B ratio with this microbial shift correlating with greater BMI gain and predicted functional enrichment in serotonin and SCFA pathways notably, features of the baseline microbiome were predictive of subsequent weight gain risk (67). Yuan et al., reported in first-episode, schizophrenia that 24 weeks of risperidone administration resulted in simultaneous increases in BMI, adverse glucose and lipid profiles, and dynamic alterations in microbiome composition (80). Extending this, Li et al. identified baseline abundance of taxa (e.g., Christensenellaceae and *Enterobacteriaceae*) as potential predictors for the development of antipsychotic-induced metabolic disturbances (81). At the taxa level, Flowers et al. (82) observe in adults with bipolar disorder that antipsychotic exposure was associated with reduced bacterial diversity and specific shifts in *Lachnospiraceae*, *Akkermansia,* and *Sutterella* with effects particularly marked in females. However, not all studies show robust compositional changes: in one cohort of acutely ill, hospitalized patients treated with olanzapine, significant weight gain occurred without major shifts in overall microbiota composition, highlighting the influence of factors such as diet, duration, and host background (83). Singh et al. synthesized evidence across multiple studies implicating antipsychotics in disruption of intestinal barrier integrity and reduction of SCFA synthesis, processes linked to observed metabolic abnormalities (84). These microbiome-mediated interactions extend beyond antipsychotics. Epidemiologic studies (85, 86) have shown that many antidepressants also possess antimicrobial properties and are associated with microbiome shifts at the population level (85–88). Rodent studies indicate that fluoxetine and other antidepressants can reduce alpha diversity and alter taxa such as *Ruminococcus* and *Adlercreutzia* (89). Lukić et al. provided striking evidence that transplantation of *Ruminococcus flavefaciens* abolished fluoxetine’s antidepressant efficacy and altered host cortical gene expression, notably downregulating neuroplasticity genes while upregulating oxidative phosphorylation genes, illustrating that gut microbes can directly influence brain molecular programs with metabolic implications (89). Collectively, these findings illustrate a bidirectional relationship in which psychotropic medications (both antipsychotics and antidepressants) can remodel the gut microbiota, while the microbiome itself modulates drug efficacy, bioavailability and downstream host metabolism. This integrated framework termed psycho pharmaco-microbiomics (90, 91) provides a mechanistic basis for interindividual variation in both therapeutic response and metabolic side effects during psychotropic treatment. These mechanistic insights are beginning to translate into microbiome targeted mitigation strategies for antipsychotic associated metabolomic disturbance. In rodents, olanzapine associated weight gain has been attenuated by antibiotics (69) and by prebiotics (64) reported that Bimuno™ galacto oligosaccharides (B GOS) attenuated olanzapine induced weight gain while increasing fecal *Bifidobacterium*. In clinical schizophrenia populations, two randomized trials reported that probiotics plus dietary fiber reduced antipsychotic associated weight gain and metabolic disturbance, with favorable effects linked to increased gut-microbial abundance (92, 93). These strategies illustrate the therapeutic potential of modifying the gut–brain–metabolic axis to minimize drug-induced metabolic complications. The underlying pathways and organ interactions are synthesized in Figure 1 and representative evidence linking drug exposure, microbiome shifts, and metabolic outcomes is summarized in Table 1.

**Table 1:** Representative studies linking psychotropic medication exposure to gut microbiome alterations and metabolic phenotypes.

Reference	Population	Medication	Microbiome method	Study design/duration	Microbiome changes	Metabolic effects
Bahr et al. (67)	Pediatric males, psychiatric, *n* = 18	Risperidone	Stool; 16S rRNA; PICRUSt	Cross-sectional (>12 mo RSP) + prospective (~10 mo)	↓ Bacteroidetes: Firmicutes; progressive decline post-RSP onset; ↑ predicted SCFA-related pathways	↑ BMI / weight gain
Morgan et al. (68)	Female mice; multiple strains + germ-free cohorts	Olanzapine	Stool; 16S rRNA (V4); molecular-tag PCR	Strain survey (10 wk. HFD ± OLZ); germ-free (7 wk. OLZ → colonization → 7 wk. OLZ); crossover (15 wk)	OLZ shifts microbiota toward obesogenic profile; microbiota-dependent β-diversity changes; OLZ shows antimicrobial activity	↑ weight gain / ↑ adiposity
Davey et al. (69)	Female Sprague–Dawley rats, *n* = 9–10/group	Olanzapine ± antibiotics	Stool; 16S rRNA (V4); 454 pyrosequencing	21 days OLZ (i.p., 2×/day) ± antibiotics (initiated 5 days prior)	OLZ alters gut microbiota; antibiotic cocktail markedly disrupts microbiota; ABX attenuates OLZ-induced changes	↓ weight gain, ↓ inflammation
Bahr et al. (63)	Female C57BL/6 J mice	Risperidone ± antibiotics / phage fraction	Stool; 16S rRNA (V4)	Chronic RSP (80 μg/day, oral) ± antibiotics or phage transfer; fecal transfer experiments	RSP alters gut microbiota; fecal transplant from RSP-treated mice reduces RMR; phage fraction alone induces metabolic shift; RSP suppresses anaerobic bacterial growth	↓ resting metabolic rate; ↑ weight gain
Yuan et al. (80)	Adults; drug-naïve; *n* = 41 completers	Risperidone	Stool; qPCR for targeted taxa	Baseline vs. 24-week RSP treatment	Baseline: ↓ *Bifidobacterium*, ↓ *E. coli*, ↓ *Lactobacillus;* ↑ *Clostridium coccoides.* Post-RSP: ↑ *Bifidobacterium*, ↑ *E. coli*; ↓ *Clostridium coccoides*, ↓ *Lactobacillus. Bifidobacterium* change correlates with weight/BMI gain	↑ weight, ↑ BMI, ↑ glucose, ↑ triglycerides, ↑ LDL, ↑ hs-CRP, ↑ HOMA-IR
Li et al. (81)	Adults; drug-naïve, *n* = 69 completers	Risperidone	Stool; 16S rRNA (V3–V4)	Baseline vs. 12- and 24-week RSP treatment	↓ Bacteroidetes; ↑ Proteobacteria; ↓ Christensenellaceae; ↑ Enterobacteriaceae; β-diversity reduction; baseline Christensenellaceae/Enterobacteriaceae predict metabolic changes	↑ BMI, ↑ glucose, ↑ HOMA-IR, ↑ triglycerides, ↑ Total-C, ↑ LDL-C, ↓ HDL-C
Flowers et al. (82)	Adults with bipolar disorder; *n* = 117	Atypical antipsychotics (AAPs)	Stool; 16S rRNA (V4)	Cross-sectional: AAP-treated vs. non-AAP	Distinct β-diversity separation; ↓ species diversity in females; ↑ Lachnospiraceae (OTU1); ↓ *Akkermansia* (OTU25), ↓ *Sutterella* (OTU32)	↑ BMI in AAP users (cross-sectional association)
Pełka-Wysiecka et al. (83)	Adults with schizophrenia; inpatients; *n* = 20	Olanzapine	Stool; 16S rRNA (V4),	7-day washout → 6-week OLZ treatment	No significant changes in gut composition or predicted function; stable individual microbiome clusters (Prevotella-dominant vs. Bacteroides/Blautia/Clostridium profiles)	↑ BMI in females only; clinical improvement
Lukić et al. (89)	Male mice (BALB/c), n per group 9–12	Antidepressants (fluoxetine, escitalopram, venlafaxine, duloxetine, desipramine)	Stool; 16S rRNA	21-day chronic antidepressant treatment	↓ richness; ↑ β-diversity; ↓ *Ruminococcus*; ↓ *Adlercreutzia*	*R. flavefaciens* attenuates duloxetine behavioral effect
Kao et al. (64)	Female Sprague–Dawley rats	Olanzapine ± prebiotic B-GOS	Stool; qPCR for targeted genera	21-day B-GOS (0.5 g/kg/day) + 14-day OLZ (10 mg/kg/day)	B-GOS ↑ *Bifidobacterium*; ↓ select Firmicutes; OLZ alone no notable microbiota change	B-GOS attenuates OLZ-induced weight gain; modulates acetate, ACC, GPR43, TNFα

RSP, risperidone; OLZ, olanzapine; AAP, atypical antipsychotic; B-GOS, Bimuno galacto-oligosaccharides; GF, germ-free; HFD, high-fat diet; SCFA, short-chain fatty acids; OTU, operational taxonomic unit; qPCR, quantitative PCR; PICRUSt, Phylogenetic Investigation of Communities by Reconstruction of Unobserved States.

## 4 Genomic insights into psychotropic-induced metabolic disturbances

While variation in microbiome composition and other molecular and environmental factors undoubtedly contribute to the metabolic sequelae of psychotropic medications, accumulating evidence demonstrates that host genetic architecture exerts a substantial and independent influence on individual susceptibility, shaping both baseline risk and drug-specific metabolic outcomes. Family and twin designs have repeatedly estimated that genetic factors account for a large fraction of antipsychotic induced weight gain, with heritability estimates commonly placed in the ~60–80% range (5, 94), aligning with the observation that susceptibility clusters within families and that early, steep weight gain trajectories often recur across treatment episodes. Conceptually, this positions psychotropic induced metabolic dysfunction as a polygenic, pathway structured adverse drug response, rather than a nonspecific side effect. Table 2 summarizes key genomic findings in psychotropic-induced metabolic disturbances, including candidate-gene and genome-wide evidence. Early pharmacogenetic efforts focused on candidate genes grounded in receptor pharmacology and appetite biology, particularly serotonergic, dopaminergic, adrenergic and leptin–melanocortin pathways, because SGAs bind 5 HT2C, H1, D2 and M3 receptors that plausibly converge on appetite, satiety and insulin regulation. Candidate associations have been reported for HTR2C (5 HT2CR) (87, 88, 95–99), DRD2 (100–106), ADRA2A (107–112) and CNR1 (113–115), alongside energy balance genes such as MC4R (116–119), LEP (120–122), and lipid regulatory programs including SREBP/SREBF (123, 124), with additional signals reported in neurodevelopmental and synaptic plasticity genes such as BDNF (125–127) and SNAP25 (125–128) (Table 2).

**Table 2:** Major genomic findings in psychotropic-induced metabolic disturbances.

Reference	Design	Duration	Method	Medications	Population	Gene/variant(s)	Key metabolic findings
(129)	GWAS	≤18 mo	SNP array	OLZ, PER, QUE, RIS, ZIP, CLZ	SCZ; EA/AA/Other; *n* = 738	MEIS2 rs1568679; PRKAR2B rs13224682; FHOD3 rs17651157/rs10502661; GPR98 rs1967256/rs11954387	MEIS2 → RIS waist/hip↑; PRKAR2B → CLZ TG↑; FHOD3 → PER TG↑
(130)	GWAS + reps	6–12 wk	SNP array	SGAs	Antipsychotic-naïve pediatrics; *n* = 139(+73/40/92)	MC4R rs489693	rs489693 → severe weight↑; TG↑; insulin↑; leptin↑
(138)	Longitudinal GWAS	≤18.7 y	SNP array + imputation	OZP, CZP, QUE, RIS, ARI, AMI, PAL	Chinese SCZ; *n* = 669	ABCG2 rs6532055; SORCS1 rs2644520; UPP2 rs115843863; KIRREL3 rs2514895; SLC2A9 rs188405603; APOA5; ZPR1; GCNT4; MAST2; CRTAC1	OZP–LDL → ABCG2; ARI–TG → SORCS1; CZP–HDL → UPP2; PAL–LDL → KIRREL3; QUE–TG → SLC2A9; TG/LDL/HDL → APOA5/ZPR1/GCNT4/MAST2/CRTAC1
(134)	GWAS (EHR biobanks)	≥3 mo	SNP arrays + imputation	All SGAs	EU *n* = 9,248; AFR *n* = 2,018; HIS *n* = 1,022	rs61900075 (TTC17/API5; EU); rs2101435 + chr2 cluster (DIS3L2/NPPC; AFR); rs12653313 (PELO–ITGA1; HIS)	EU → TTC17/API5–MetS; AFR → DIS3L2/NPPC–MetS; HIS → PELO–ITGA1; MR: BMI → MetS↑
(135)	GWAS + WES	8 wk	SNP array + WES	RIS	Han Chinese SCZ; GWAS *n* = 189; WES *n* = 222	GRM7 rs141134664/rs57521140/rs73809055; PRMT3 cluster; USP48; SGCZ; DOCK5; HTATIP2; PACC1 rs117462017; SPTBN1 rs2271323/rs2271326; HMGXB3; GPR12; MAP2K3	GRM7 → RIS response; PRMT3/USP48/SGCZ clusters; WES rare variants: SPTBN1/PACC1/HMGXB3; gene-based → GPR12, MAP2K3
(136)	GWAS	≤6 mo	SNP array	Psychotropics (APs, mood stabilizers, antidepressants)	Psychiatric cohort; EU; *n* = 1,135	rs7736552 (MAN2A1); rs11074029 (SLCO3A1); rs117496040 (DEFB1); rs7647863 (IQSEC1)	rs7736552 → BMI slope; rs11074029 → BMI slope (6 mo); rs117496040 → BMI change; rs7647863 → BMI change (3 mo)
(137)	GWAS + validation	4–8 wk	SNP array + imputation	OLZ, RIS, QUE, ARI, ZIP	Han Chinese SCZ discovery *n* = 1936; validation *n* = 534; CATIE *n* = 630	PEPD rs10422861; PTPRD rs3824417; PEPD rs3786900 (coloc with T2D)	PEPD rs10422861 → AIWG↑; PTPRD rs3824417 → AIWG↑; AIWG↔lipid changes (LDL/TG↑); PRS AIWG/T2D → weight gain
(97)	Candidate-gene	3 mo	Genotyping	OLZ, RIS	Female SCZ; Caucasian; *n* = 101	5-HT2C rs3813929 (−759C/T); MDR1 rs2032582 (G2677T); rs1045642 (C3435T)	5-HT2C − 759 T → waist↑; glucose↑; TG↑; MDR1 2,677 T → OLZ glucose↑; MDR1 3,435 T → OLZ glucose↑ (borderline)
(103)	Candidate-gene	≥4–6 wk	qPCR genotyping (TaqMan)	RIS	Indian SCZ; *n* = 289 (prolactin *n* = 102; weight *n* = 97)	DRD2 rs1799732 (−141C Ins/Del); 5-HTR2C rs3813929 (−759C > T)	DRD2–141 Ins/Del/Del → prolactin↑ (OR ≈ 10.45); 5-HTR2C − 759C > T → no assoc.; weight/EPS → no assoc.; EPS 36.7%, hyperprolactinemia 87.2%, weight gain 53.6%
(111)	Candidate-gene	≥3 mo	RFLP genotyping (MspI)	OLZ	Korean SCZ; *n* = 62	ADRA2A rs1800544 (−1291C > G)	G allele → severe weight gain (>10%) OR ≈ 2.58; genotype → weight change *p* = 0.028
(113)	Prospective follow-up (nested case–control)	6 mo	Targeted sequencing (38 SNPs)	All antipsychotics	European; *n* = 137	CNR1 rs1049353; INSIG2 rs7566605	CNR1 rs1049353 (dominant) → weight gain↓ (OR ≈ 0.41); INSIG2 rs7566605 (recessive) → weight gain↑ (OR ≈ 3.03); OLZ → risk↑; QTP → risk↓
(118)	Candidate-gene	≤14 wk	TaqMan SNP genotyping	CLZ, OLZ, RIS, HAL	SCZ; mixed ancestry; *n* = 224	MC4R rs8087522; rs17782313; rs11872992	rs8087522 A-allele → CLZ weight gain↑ (EU subgroup; *p* = 0.027); other SNPs → no sig
(122)	Candidate-gene	≤14 wk	TaqMan SNPs	CLZ, OLZ, RIS, HAL	SCZ/SA; mixed; *n* = 181	LEP rs7799039; rs10954173; rs3828942; LEPR rs1327120; rs1137101	LEP haplotype (rs7799039G–rs10954173G–rs3828942G) → AIWG↑; rs7799039 G → AIWG↑; rs3828942 G → AIWG↑; LEPR rs1137101/rs1327120 → no assoc
(126)	Candidate-gene	≤14 wk	TaqMan genotyping	CLZ, OLZ, HAL, RIS	EU + AFR; *n* = 188	IL-1β rs4849127; rs16944; rs1143634; IL-2 rs2069772/rs2069776 etc.; IL-6 rs2069837; BDNF Val66Met	IL-1β rs4849127/rs16944/rs1143634 → AIWG↑; BDNF Val66Met Met → AIWG↓; IL-1β × BDNF and IL-6 × BDNF epistasis → risk↑
(128)	Case–control	≥10 wk	MassARRAY genotyping	HAL, OLZ, RIS	Han Chinese; SCZ *n* = 3,243 + controls 6,154; AIWG subset *n* = 2,128	SNAP25 rs6039769; rs3787283; rs3746544	rs6039769 CC → AIWG↑ across HAL/OLZ/RIS (*p* < 0.001); no effect on PANSS; rs3787283/rs3746544 → no AIWG

AIWG, antipsychotic-induced weight gain; APs, antipsychotics; ARI, aripiprazole; AMI, amisulpride; APZ/OZP, olanzapine; CATIE, Clinical Antipsychotic Trials of Intervention Effectiveness; CLZ, clozapine; CZP, clozapine; EA, European ancestry; AFR, African ancestry; HIS, Hispanic ancestry; EPS, extrapyramidal symptoms; EU, European; GWAS, genome-wide association study; HAL, haloperidol; HDL, high-density lipoprotein; LDL, low-density lipoprotein; MetS, metabolic syndrome; PAL, paliperidone; PER, perphenazine; QTP/QUE, quetiapine; RIS, risperidone; SA, schizoaffective disorder; SCZ, schizophrenia; SGAs, second-generation antipsychotics; SNP, single-nucleotide polymorphism; TG, triglycerides; WES, whole-exome sequencing. Genetic associations listed correspond to reported significant or replicated findings in their respective studies; effect directions (↑/↓) indicate increased or decreased metabolic measures, respectively.

Genome-wide association studies (GWAS) have therefore been increasingly used to map the genetic architecture of psychotropic induced metabolic changes across multiple phenotypes. One of the earliest and most comprehensive genome-wide pharmacogenomic efforts was conducted by Adkins et al. (129) using the CATIE genomic subsample. In 738 schizophrenia patients genotyped for ~492 K SNPs, the authors modeled 12 quantitative metabolic side-effect indicators spanning antipsychotic induced changes in weight and body size measures, blood lipids, glucose and HbA1c, blood pressure and heart rate. Using a prespecified false discovery approach (designed so that ~10% of significant findings would be false discoveries), Adkins et al. identified 21 SNPs meeting their criterion. Their top signal implicated a SNP in MEIS2 that mediated risperidone associated changes in hip circumference (*q* = 0.004) and signaled for waist circumference (*q* = 0.055). Additional genome wide signals were reported in or near PRKAR2B, GPR98, FHOD3, RNF144A, ASTN2, SOX5 and ATF7IP2, among others. While replication and functional validation were explicitly highlighted as necessary, the study was influential for shifting the field from receptor candidate hypotheses toward broader pathway discovery and for emphasizing that antipsychotic metabolic toxicity is not a single phenotype but a constellation (adiposity, glycaemia, lipids, haemodynamics) that may map to overlapping and partially distinct genetic determinants (Table 2). Subsequent GWAS work has helped anchor certain loci in biologically coherent appetite and energy homeostasis pathways, particularly the leptin-melanocortin axis. A key pediatric signal emerged from the first GWAS explicitly focused on antipsychotic related weight gain in youth: in patients ≤19 years (*n* = 139) treated with quetiapine, risperidone, olanzapine or aripiprazole, a SNP downstream of MC4R (rs489693) was identified (130) and later supported by independent studies implicating variants near MC4R (117–119). This locus is mechanistically plausible: leptin activates hypothalamic POMC neurons, leading to α MSH production, which suppresses food intake through MC4R signaling, while AgRP antagonizes MC4R; serotonergic 5 HT2C signaling intersects upstream of this axis, and downstream mediators such as BDNF have been proposed to participate in MC4R linked appetite regulation (131–133).

More recent pharmacogenomic studies have moved beyond “weight only” endpoints toward drug specific lipid and BMI trajectories, which is crucial for a metabolomic framing. In a large longitudinal Chinese schizophrenia cohort, Wong et al. (134) performed GWASs targeting SGA induced lipid and BMI changes across seven SGAs (olanzapine, clozapine, quetiapine, risperidone, aripiprazole, amisulpride and paliperidone). Their dataset combined genotypes (*N* = 669) with dense clinical follow-up (19,316 prescription records and thousands of metabolic measurements per outcome; follow up to 18.7 years, mean 5.7 years). Using linear mixed models to estimate patient specific random effects for each SGA and outcome, they reported five SNPs reaching *p* < 5 × 10^−8^ before multiple testing correction, including rs6532055 in ABCG2 linked to olanzapine induced LDL change, rs2644520 near SORCS1 linked to aripiprazole induced triglyceride change, rs115843863 near UPP2 linked to clozapine induced HDL change, rs2514895 near KIRREL3 linked to paliperidone induced LDL change, and rs188405603 in SLC2A9 linked to quetiapine induced triglyceride change. While these SNPs did not survive the authors’ stringent Bonferroni correction across 160 GWAS analyses, they passed FDR correction at 0.2, and gene-based analyses identified genome wide significant genes after Bonferroni correction, ABCG2, APOA5, ZPR1, GCNT4, MAST2 and CRTAC1, along with gene sets associated with SGA induced metabolic side effects. The study’s design is notable for modeling agent specific metabolic effects at scale, strengthening the concept that different SGAs may engage partially separable genetic architectures for lipid versus adiposity outcomes. Important complementary direction is to interrogate metabolic syndrome as a composite phenotype that better captures the clustered metabolomic disturbance observed clinically under SGAs. El Rouby et al. (134), conducted a GWAS of SGA induced metabolic syndrome (SGA MetS) using EHR linked biobanks (BioVU and BioMe) and additionally applied Mendelian randomization (MR) to evaluate the contribution of individual MetS components (BMI, fasting glucose, blood pressure, HDL and triglycerides) to SGA MetS. In European ancestry meta-analysis across the two biobanks (*N* = 9,248), they identified a genome wide signal on chromosome 11 (rs61900075, *β* = −0.27, SE = 0.05, *p* = 1.6 × 10^−8^) and reported additional suggestive association within the PELO-ITGA1 locus on chromosome 5, including signals in Hispanic ancestry participants within BioMe. In African ancestry participants (*N* = 2,018), multiple genome wide signals were mapped to NPPC–DIS3L2 on chromosome 2. Their MR analysis further supported BMI as a key driver within the syndrome architecture: genetically predicted higher BMI increased the odds of SGA MetS (inverse variance weighted OR = 1.2, 95% CI 1.1–1.4, *p* = 0.002), with concordant sensitivity analyses, providing genetic evidence consistent with BMI acting as an amplifier of broader metabolomic liability under SGA exposure rather than merely a correlated marker.

A parallel genomic literature has focused on treatment response, which is not itself a metabolic endpoint but is relevant in multi omics frameworks because it determines exposure duration and polypharmacy patterns and may share signaling biology with metabolic toxicity (for example via GPCR networks). Zhao et al. (135) combined SNP microarray GWAS (*n* = 189) and whole exome sequencing (WES; *n* = 222) in multi center Chinese cohorts to identify genetic correlates of 8-week risperidone response. They reported signals implicating GPCR-related pathways, illustrating mixed common/rare architecture relevant to exposure and downstream metabolic risk. Additional evidence comes from Sjaarda et al. (136) who conducted a GWAS in the Swiss PsyMetab cohort (*N* = 1,135) to examine BMI evolution during early psychotropic treatment. They identified four novel loci at genome wide significance (rs7736552 near MAN2A1, rs11074029 in SLCO3A1, rs117496040 near DEFB1, and rs7647863 in IQSEC1), with replication in UK Biobank participants under psychotropic exposure. A complementary large GWAS in Han Chinese patients (137) identified genome-wide loci (PEPD, PTPRD) for antipsychotic-induced weight gain and integrated PRS and MR analyses linking early lipid changes to weight gain (Table 2). Taken together, available genomic studies support several principles that are directly relevant to psychotropic induced metabolomic disturbance. First, susceptibility appears polygenic, with individual loci often exerting small effects and requiring large, harmonized cohorts for reproducible discovery. Second, genetic architecture is likely agent specific and phenotype specific: variants that modulate olanzapine associated LDL trajectories [for example, ABCG2 (138)] may not predict aripiprazole associated triglyceride change (near SORCS1), and loci mapping to appetite circuitry (MC4R region) may not capture lipid dominant or composite MetS phenotypes. Third, clinical translation will probably depend less on single variants and more on polygenic risk models and integrative predictors that combine host genomics with other omics layers (microbiome, metabolomics/lipidomics, endocrine markers) and exposure context (age, sex, baseline BMI, prior antipsychotic exposure and treatment duration). In this sense, genomics provides the inherited “risk scaffold” on which psychotropic exposure builds metabolomic phenotypes, helping to explain why the same pharmacological perturbation yields highly divergent metabolic outcomes across individuals.

## 5 Epigenomic regulation as a mediator of psychotropic-induced metabolomic disturbance

While germline genetic variants establish a baseline for metabolic risk, epigenomic mechanisms add a dynamic, environment-sensitive regulatory layer, adapting gene expression to pharmacological exposures and lifestyle factors in real time. DNA methylation, histone modification, and non-coding RNA regulation form the principal axes of this modifiable interface between the genome, exposome, and phenotype, contributing significantly to both the onset and persistence of psychotropic-induced metabolic dysfunction, including IR. Recent epigenome-wide association studies (EWAS) in psychiatric populations undergoing SGA treatment have identified both widespread and locus-focused changes in DNA methylation. Dubath et al. (11) performed a prospective observational study of 79 patients initiating a weight-gain-inducing psychotropic, assessing methylation at baseline and 1 month using the Illumina EPIC BeadChip. They observed a global methylation increase after the first month, with more pronounced changes in patients maintaining stable weight. Notably, epigenome-wide significant methylation changes occurred at 52 loci across the cohort, with one locus (cg12209987) showing a significant association with both increased methylation and early weight gain (≥5% increase). While the primary epigenome-wide association analysis did not reveal direct links between metabolic change and methylation at stringent significance levels, Mendelian randomization analyses implicated baseline methylation at cg11622362 as potentially causal for glycaemic change, providing rare mechanistic support for an epigenetic contribution to acute psychotropic-induced metabolic shifts.

Individual candidate and pathway-focused methylation studies further highlight the specificity of these changes. Hypermethylation of promoters for genes essential to insulin signaling (e.g., INS, IRS1, PIK3R1) (139–142), lipid metabolism (PPARG, PPARA, SREBF1/2) (143, 144), adipokine regulation (LEP, ADIPOQ) (145), and anti-inflammatory cytokines (ABCG1) (146) has been described in both blood and metabolically active tissues, frequently correlating with *in situ* gene silencing and reduced insulin sensitivity, and sometimes predating overt metabolic dysfunction. Evidence is accruing that these modifications are not limited to established side effects; for instance, olanzapine-induced IR in both animal and clinical models has been linked to increased DNA methylation of the GLUT1 promoter, hypermethylation at key loci within the hepatic and muscular insulin transduction cascade, and global hypermethylation in peripheral and hepatic tissues (147, 148). Recent trials in healthy volunteers demonstrate that olanzapine can alter methylation in a suite of insulin sensitivity related genes, including PPARGC1A, PRKAR1B, and RPTOR, in parallel with experimentally induced reductions in insulin sensitivity (149).

Histone modifications constitute a second major regulatory axis. Valproic acid, a mood stabilizer with well-established HDAC-inhibitory properties, induces widespread histone acetylation and de-repression of gene networks controlling energy metabolism, mitochondrial function, and inflammation (150). With antipsychotic exposure, histone acetylation and methylation changes have been described at both activating (e.g., H3K4me3, H3K27ac) and repressive (e.g., H3K27me3) marks in metabolic gene promoters, often preceding or tracking the onset of key side effects. Specifically, Su et al. found that olanzapine in female rats induced H3K4me2 increases and H3K9me3 decreases in hepatic tissue, modifications plausibly linked to perturbation of metabolic gene expression and IR. The co-administration of beta-histine, a compound known to mitigate olanzapine-induced weight gain, reversed some of these histone changes, reinforcing a mechanistic link between histone status, drug interaction, and metabolic phenotype (151, 152). Concordantly, investigations in neural tissue (hippocampus) and brown adipose tissue show olanzapine-induced modulation of histone acetylation and methylation states, affecting both thermogenesis and central metabolic control (153, 154).

Non-coding RNA (ncRNA) regulation is increasingly recognized as a critical fine tuner of metabolic gene expression in the pharmacological context. Antipsychotic therapy has been associated with altered levels of multiple microRNAs (e.g., miR-22, miR-181b, miR-339, miR-193, miR-223, miR-544), long non-coding RNAs (lncRNAs, such as Gomafu), and small nucleolar RNAs, each with roles in insulin sensitivity, β-cell function, and adipocyte biology (155–158). These ncRNAs regulate the translation of key metabolic effectors (e.g., insulin receptor, GLUT4) and may integrate peripheral metabolic adaptation with central neural circuitry impacted by psychotropics. For instance, reductions in miR-29, miR-375, and miR-185-5p have been linked with more severe IR following antipsychotic exposure. Further insights have come from additional EWAS analyses. For example, differential methylation in the fatty acyl CoA reductase 2 (FAR2) gene have been associated with IR (159), and methylation at a cadherin-like 22 (CDH22) gene locus has been linked to metabolic syndrome (160). The gut microbiome may also modify host epigenomic regulation during psychotropic exposure. Dysbiosis can reduce SCFA production, especially butyrate, which naturally acts as an endogenous HDAC inhibitor. As a result, psychotropic-induced dysbiosis may indirectly decrease beneficial histone acetylation and anti-inflammatory gene expression, thereby promoting a metabolic environment predisposed to IR and steatosis. Restoration of SCFA production, via diet, prebiotics, or targeted microbial interventions, can partially reverse maladaptive epigenetic repression in preclinical models, suggesting a plausible molecular link from environmental intervention to improved metabolic risk (8, 161, 162). Clinically, the high plasticity and reversibility of these epigenetic processes offer promising avenues for intervention. Dietary fiber enrichment, targeted modulation of the microbiome, and potentially direct employment of epidrugs could augment strategies to prevent or ameliorate psychotropic-induced metabolic complications. The integration of EWAS, methylome, and histone profiling, with genetic, transcriptomic, and comprehensive clinical datasets, holds substantial promise for advancing personalized metabolic risk prediction and tailored intervention in patients requiring long-term psychopharmacotherapy. Key epigenomic studies linking psychotropic exposure to metabolic outcomes are summarized in Table 3.

**Table 3:** Epigenomic evidence linking psychotropic exposure to metabolic disturbances.

Reference	Design	Methodological platform	Medications investigated	Population/model	Epigenetic/transcriptomic marker(s)	Principal findings
(11)	Prospective observational EWAS	Illumina MethylationEPIC (850 K CpGs)	SGAs, lithium, valproate, mirtazapine	Psychiatric cohort (*n* = 79)	Global DNAm↑; 52 CpGs altered; cg12209987↑; cg11622362 implicated	Treatment-associated DNAm increase; cg12209987 predicts early weight gain; cg11622362 methylation causally linked to glycaemia
(146)	Multi-omics integration (GWAS, WES, EWAS, CRISPR)	EPIC DNAm array + sequencing + functional assays	Antipsychotics (esp. olanzapine)	SCZ cohorts (GWAS 939; WES 364; EWAS 268)	ABCG1 hypermethylation; DMR at chr21	ABCG1 methylation associates with dyslipidaemia; CRISPR validation shows causal lipid accumulation; PTPN11↑ induces insulin resistance
(147)	*In vivo* antipsychotic methylome profiling	MeDIP-chip genome-wide DNAm	Olanzapine	Rat hippocampus, cerebellum, liver	Tissue-specific hyper- and hypomethylation (≈1,100–1,300 genes)	Olanzapine perturbs dopamine–DARPP32, cAMP and ephrin signaling; liver lipid-metabolism pathways affected; weight gain observed
(148)	Ex vivo PBMC stimulation	18F-FDG uptake; qRT-PCR; Western blot; bisulfite sequencing	Olanzapine; aripiprazole	Human PBMC	CpG-34 hypermethylation in GLUT1 promoter	Olanzapine reduces FDG-uptake, PDH activity and AMPKα; aripiprazole increases FDG-uptake; both ↑GLUT1 protein
(147)	miRNA profiling and miRNA–mRNA network modeling	Illumina miRNA array; Affymetrix mRNA array; Bayesian network analysis	Haloperidol, olanzapine, clozapine	C57BL/6 mouse brain	Drug-specific miRNA signatures (haloperidol: 6↓; olanzapine: 5↓; clozapine: 5↑)	Olanzapine and clozapine enrich metabolic pathways (lipid, carbohydrate metabolism, obesity); olanzapine-specific miRNA regulatory modules
(155)	Clinical case–control + longitudinal expression	qRT-PCR	Mixed antipsychotics	SCZ patients (*n* = 35) + controls (*n* = 49)	lncRNA Gomafu/MIAT	Elevated in untreated SCZ; further ↑ after 12 weeks of antipsychotics; expression change appears disease-related rather than treatment-responsive
(156)	Clinical longitudinal plasma miRNA analysis	qRT-PCR (9 SZ-associated miRNAs)	Olanzapine, quetiapine, ziprasidone, risperidone	SCZ patients (*n* = 20 baseline; *n* = 17 follow-up) + controls (*n* = 20)	miRNA-181b, miR-30e, miR-34a, miR-7	miR-181b significantly down-regulated after treatment; magnitude of reduction predicts negative-symptom improvement (OR = 11.28); candidate plasma biomarker for treatment response

APD, antipsychotic drug; AMPK, AMP-activated protein kinase; BDNF, brain-derived neurotrophic factor; CpG, cytosine–phosphate–guanine dinucleotide; CRISPR, clustered regularly interspaced short palindromic repeats; DNAm, DNA methylation; DMR, differentially methylated region; EWAS, epigenome-wide association study; FDG, fluorodeoxyglucose; GLUT1, glucose transporter 1; GWAS, genome-wide association study; lncRNA, long non-coding RNA; MeDIP, methylated DNA immunoprecipitation; miRNA, microRNA; MR, Mendelian randomization; PBMC, peripheral blood mononuclear cell; PDH, pyruvate dehydrogenase complex; qRT-PCR, quantitative reverse-transcription PCR; SCZ, schizophrenia; SGA, second-generation antipsychotic; WES, whole-exome sequencing.

## 6 Transcriptomic dynamics in psychotropic medication–induced metabolomic disturbance and insulin resistance

A rapidly expanding transcriptomic literature has begun to illuminate the molecular and cellular mechanisms by which psychotropic drugs, including antipsychotics and mood stabilizers, drive widespread reprogramming of metabolic gene networks, predisposing many patients to IR, dyslipidemia, and altered energy balance. Most studies employ genome-wide RNA sequencing or high-resolution microarray profiling of whole blood or patient-derived cellular models, with additional focus on mechanistic roles for non-coding RNAs. A paradigm-shifting example is the work by Sainz et al. (163), who performed transcriptomic profiling on peripheral blood from first-episode psychosis patients undergoing second-generation antipsychotic treatment. Remarkably, before treatment commenced, distinct gene expression patterns differentiated eventual clinical responders from non-responders, suggesting that a patient’s baseline transcriptomic state may predict both therapeutic and metabolic susceptibility to antipsychotics. After 12 weeks, this divergence became more pronounced, with significant upregulation in responders of genes related to neutrophil degranulation and lipid metabolism. The identification of a four-gene signature (including SLC9A3, HMOX1, and SLC22A16) as a highly accurate predictor of clinical response exemplifies the translational utility of transcriptomic risk stratification.

A follow-up study by the same group (164) demonstrated that weight gain during antipsychotic treatment was accompanied by reproducible shifts in expression of dozens of genes, mainly mapped to immune and inflammatory pathways. Intriguingly, they documented pronounced sex differences: men exhibited broadened immune and neutrophil activity gene upregulation with weight gain, while women showed fewer changes, largely enriched for interferon signaling and immune regulation. These findings highlight the necessity of sex-stratified analytic approaches and suggest differential transcriptomic vulnerability to antipsychotic-induced metabolic side effects. Further research into acute drug effects, such as the study by Mas et al. (165), revealed that patients who developed extrapyramidal symptoms during short-term antipsychotic exposure displayed unique transcriptomic profiles, including upregulation of genes involved in insulin receptor signaling, lipid modification, autophagy, and innate immune function. These results reinforce the close interplay between metabolic, immune, and neurologic adaptation at the transcriptomic level in the face of psychotropic challenge. The value of transcriptomics extends to severe adverse event prediction. In schizophrenia patients who experienced clozapine-induced agranulocytosis (166) and others identified dynamic changes in apoptosis-related gene expression, including normalization after clinical intervention, demonstrating the potential of gene expression profiling for tracking both susceptibility and resolution of serious drug toxicities. Transcriptomic mapping also offers critical mechanism and predictive insights in mood stabilizer therapy. A recent study have uncovered gene expression modules enriched for energy metabolism and mitochondrial function that predict lithium response in bipolar disorder, while neuronal modeling has shown that lithium’s effect on synaptic and energetic gene normalization may be restricted to responders (167).

In sum, transcriptomic profiling in patients exposed to psychotropic drugs reveals a highly responsive molecular landscape and captures early events in metabolic pathway reorganization, often before clinical toxicity emerges. Recurring signatures include the upregulation of immune/inflammatory genes, metabolic stress markers, and those regulating mitochondrial and lipid metabolism. Critically, these expression changes are frequently sex- and syndrome-specific and, in combination with clinical and other -omic data, hold promise for development of early biomarkers for prediction and monitoring of psychotropic-induced metabolic risk. Ongoing advances in multi-omics, longitudinal research frameworks are poised to drive the translation of transcriptomic markers into individualized strategies for metabolic surveillance and intervention in psychiatric care.

## 7 Metabolomic signatures in psychotropic medication–induced metabolomic disturbance and insulin resistance

Metabolomic profiling offers an unprecedented molecular window into the cumulative impact of psychotropic therapy on metabolic homeostasis. This “downstream” omics approach provides a functional readout of gene–environment-microbiome interactions, reflecting the rapid onset, multidimensional, and agent specific biochemical perturbations that underlie psychotropic-induced IR, dyslipidemia, weight gain, and systemic inflammation.

Lenski et al. (168) performed targeted metabolomic profiling in 62 patients initiating psychotropic medications with known metabolic liability. Within just 1 month, they identified a panel of 19 short-term plasma metabolites that predicted weight gain at 3 months. These included multiple amino acids (glutamine, asparagine, histidine, ornithine), acylcarnitines (hexanoylcarnitine, octanoylcarnitine, tetradecenoylcarnitine, butyrylcarnitine), carboxylic acids (3-hydroxybutyric acid, methylsuccinic acid, malonic acid), catecholamines (dopamine, normetanephrine), nucleosides (guanosine), pyridine (trigonelline), tetrapyrrole derivatives (biliverdin), and key kynurenine pathway metabolites (kynurenine, tryptophan, kynurenic acid, 3-hydroxykynurenine, xanthurenic acid, anthranilic acid), with the kynurenine/tryptophan ratio standing out as a robust prospective indicator of ongoing metabolic risk. Hexanoylcarnitine, biliverdin, and kynurenine were specifically associated with longitudinal BMI increase, while confirmation in an independent cohort reinforced the reliability of kynurenine pathway shifts. Such panels highlight the mechanistic interplay of disrupted amino acid metabolism, mitochondrial β-oxidation (acylcarnitine elevation), neuroinflammation, and oxidative stress in the emergence of psychotropic-induced metabolic effects. Untargeted metabolomics studies further support these findings. Most perturbed metabolite classes in patients receiving antipsychotics, mood stabilizers, or high-risk antidepressants include carboxylic acids (encompassing amino acids, TCA cycle intermediates, fatty acids), keto acids (α-ketoglutaric acid, pyruvic acid), and organic acids. Increased levels of branched-chain amino acids (leucine, isoleucine, valine), aromatic amino acids (phenylalanine, tyrosine), and β-alanine are consistently found. These connect to both the pathophysiology of IR and the altered neurotransmitter and synaptic plasticity thought to underline both clinical efficacy and adverse effects. Elevated glyceric acid and 3-hydroxybutyric acid reflect impaired energy homeostasis and fatty acid oxidation, while increased methylsuccinic acid and malonic acid point to both mitochondrial stress and disrupted glucose handling. Shifts in organonitrogen compounds (trimethylamine N-oxide, TMAO), organooxygen compounds (glucose, glucuronic acid), and organic sulfonic acids (taurine) are repeatedly linked to altered microbiome-host communication, inflammation, and psychiatric treatment response (169). One of the most consistent findings across psychotropic agents, particularly with SGAs, is a preferential shift in tryptophan metabolism away from serotonin synthesis toward the kynurenine pathway, with resultant elevations in kynurenine/tryptophan ratio and associated accumulation of kynurenic acid, 3-hydroxykynurenine, xanthurenic acid, and anthranilic acid. These metabolites reinforce the mechanistic links between neuroinflammation, immune modulation, and disturbances in central and peripheral metabolic homeostasis, strengthening the molecular basis connecting psychiatric efficacy, adverse metabolic effects, and the gut–brain axis (170).

Branched-chain amino acids (BCAAs), particularly leucine, have emerged as influential metabolite features in psychiatry and metabolic research. In our metabolomics comparison of bipolar and schizophrenia patients treated with second-generation antipsychotics (SGAs), leucine was notably elevated. Leucine, along with valine and isoleucine, serves as a precursor for glutamate synthesis in the brain, which is essential for neuronal function (171). Beyond this neurochemical role, BCAAs are strongly implicated in obesity and diabetes models, highlighting their relevance to SGAs’ well-documented metabolic side effects (172). A study by Wang et al. demonstrated that six BCAAs, including leucine, predict the development of diabetes, reinforcing their biomarker potential (173). Within another cohort (174), L-leucine was higher in schizophrenia subjects on SGAs, consistent with prior findings of elevated leucine in cerebrospinal fluid of schizophrenia patients, although these were not replicated peripherally in clozapine-treated individuals (175). Amino acid supplementation has also shown promise in bipolar disorder, improving manic symptoms acutely, suggesting therapeutic implications (176). Taken together, BCAA metabolism may represent a critical pathway for understanding both the efficacy and adverse metabolic effects of SGAs, such as clozapine, olanzapine, risperidone, and quetiapine, and could gain further prominence with the integration of microbiome research in psychiatry.

## 8 Proteomics in psychotropic medication–induced metabolic dysfunction

Proteomics provides a critical mechanistic link between psychotropic exposure and metabolic phenotypes by capturing the functional molecular effectors, enzymes, transporters, signaling intermediates, and stress-response proteins, whose abundance and post-translational modifications (PTMs) directly regulate insulin sensitivity, mitochondrial metabolism, inflammatory tone, and inter-organ communication (177). This layer is particularly informative in psychotropic-induced metabolic liability, where clinically relevant changes often arise early after treatment initiation and are driven by post-transcriptional and PTM-dependent mechanisms not reliably inferred from transcriptomic data. Within integrative multi-omics frameworks, proteomics thus bridges upstream regulatory variation with downstream biochemical and clinical outcomes.

Quantitative brain proteomics demonstrates that psychotropic agents can reprogram neuroenergetic pathways beyond primary receptor pharmacology. In a controlled mouse model, chronic paroxetine exposure induced coordinated hippocampal proteome remodeling consistent with a shift toward aerobic glycolysis, reduced tricarboxylic acid cycle flux, and altered redox balance (178). Proteomic downregulation of lactate dehydrogenase B provided a mechanistic explanation for discordant metabolite patterns, while signatures of astrocyte-linked carbohydrate handling, neuron–glia metabolic coupling, purine metabolism, and inhibitory neurotransmission implicate protein-level pathways relevant to delayed antidepressant efficacy and neuroendocrine control of systemic metabolism (178). Independent studies showing SSRI-induced reductions in astrocytic glycogen with increased glycolytic output support this model (179), and integration with metabolomics enabled nomination of myo-inositol as a candidate peripheral correlation of central metabolic remodeling (178).

In peripheral tissues, proteomic profiling consistently implicates disruption of insulin signaling and mitochondrial protein networks, particularly with higher-liability second-generation antipsychotics. Alterations in IRS–PI3K–AKT signaling, GLUT4-dependent glucose transport, and proteins governing β-oxidation, tricarboxylic acid cycle flux, and oxidative phosphorylation align with metabolomic signatures of metabolic inflexibility and lipid oversupply. Concurrent activation of inflammatory and cellular stress programs, including endoplasmic reticulum stress pathways that impair insulin signaling via PTM-dependent mechanisms, frequently precedes overt clinical metabolic deterioration, underscoring the sensitivity of proteomics for early risk detection (1, 180).

A key translational strength of proteomics lies in its ability to quantify circulating mediators of inter-organ communication, including adipokines, hepatokines, myokines, lipoprotein-associated proteins, and inflammatory factors (8). Longitudinal plasma proteomics, particularly when integrated with metabolomic and microbiome-derived functional data, enables earlier risk stratification and pathway-informed monitoring than conventional clinical measures alone (181). Moreover, resolution of dynamic PTM states identifies mechanistically actionable nodes for targeted intervention and response tracking, supporting a shift toward individualized prevention of psychotropic medication–induced metabolic dysfunction.

## 9 Nutrition and lifestyle in psychotropic-induced metabolic dysfunction and insulin resistance

Nutrition and lifestyle are central to optimizing mental health outcomes and ensuring the safety and efficacy of psychopharmacological treatments. Dietary habits affect drug absorption, metabolism, and pharmacodynamics and can mitigate or exacerbate medication-related adverse effects. Among dietary patterns, the Mediterranean diet stands out for its profound benefits in supporting both physical and mental health during psychotropic therapy (Table 4).

**Table 4:** Dietary patterns, supplements, mechanisms, and psychotropic interfaces.

Item	Representative components/sources	Core nutritional elements/supplements	Mechanistic basis (per chapter)	Psychotropic interface	Clinical implications	Key evidence (from chapter)
Mediterranean diet	Fruits, vegetables, whole grains, fish, nuts, olive oil; moderate wine	Omega-3 (EPA/DHA), polyphenols, fiber, antioxidants	↓ systemic inflammation; ↓ oxidative stress; complex carbs stabilize glycemia; omega-3 s improve membrane fluidity & serotonin receptor function	SSRIs/SNRIs, mood stabilizers, antipsychotics	Enhances antidepressant outcomes; mitigates antipsychotic-related metabolic risk; supports adherence & relapse reduction	(182–185, 187)
Ketogenic diet	High-fat, very low-carb (avocado, nuts, oils)	Healthy fats; monitor Ca/Vitamin D	Ketosis → neuroprotective; ↓ inflammatory markers; potential alteration of CYP-mediated metabolism	Mood stabilizers (bipolar), antipsychotics (monitoring)	Adjunct in treatment-resistant bipolar; monitor liver enzymes, bone health; consider modified keto w/ adequate protein	(192–194)
Vegetarian / Vegan diet	Plant-based (legumes, whole grains, nuts, seeds, vegetables, fruits)	High fiber & antioxidants; supplement B12; marine-equivalent omega-3 (algae EPA/DHA)	Anti-inflammatory profile; plant ALA → limited conversion to EPA/DHA; possible lower availability of tryptophan/tyrosine precursors	SSRIs/SNRIs, mood stabilizers	Cardiometabolic benefit; prevent B12/omega-3 deficiency to maintain antidepressant efficacy & cognition	(195, 196, 198)
Vitamin D	Sunlight; fortified dairy; fatty fish	Vitamin D	Brain receptor distribution; regulates gene expression for dopamine-related enzymes	SSRIs	Adequate status associated with more favorable SSRI response; consider supplementation in low-sunlight settings	(189)
B-vitamins (B6, B12, folate)	Fish, poultry, leafy greens, legumes, fortified grains	B6 (cofactor), B12, folate	Cofactors for serotonin, dopamine, GABA synthesis; homocysteine regulation; neuronal integrity	SSRIs, antipsychotics, mood stabilizers	Assess/supplement in restrictive diets/older adults to support efficacy & cognition	(188, 200)
Magnesium	Leafy greens, nuts, seeds, whole grains	Magnesium	Modulates NMDA; supports GABAergic tone; mood & sleep stabilization	Anxiolytics, antidepressants	Adjunct for anxiety/depression; potential improvement of tolerability	(190, 201)
Zinc	Meat, shellfish, legumes, seeds, nuts	Zinc	Supports neuroplasticity; antioxidant; NMDA regulation	SSRIs (treatment-resistant depression)	Low zinc linked to poorer response; adjunct zinc associated with symptom reduction	(191, 202, 203)

The Mediterranean diet, recognized for its cardiovascular and neuroprotective roles, emphasizes fruits, vegetables, whole grains, fish, nuts, olive oil, and moderate wine intake (182). This nutrient-dense pattern provides omega-3 fatty acids, antioxidants, fiber, and polyphenols, which reduce inflammation and improve mental wellbeing (183). High antioxidants and omega-3 intake are associated with decreased inflammation, improving outcomes for those treated with antidepressants and mood stabilizers (184). Additionally, complex carbohydrates help stabilize blood sugar, mitigating metabolic side effects of antipsychotics and supporting mood stabilization. Omega-3 fatty acids from fish improve serotonin receptor function and can potentiate SSRI and SNRI efficacy, benefiting patients with mood disorders (185). These glucose-regulating effects are especially advantageous for older adults with mental health conditions or metabolic comorbidities, such as diabetes, by minimizing weight gain and IR, common sequelae of antipsychotic pharmacotherapy. In younger adults, the mood-stabilizing effects of omega-3-rich foods further support brain health (186). Studies in patients with schizophrenia show that Mediterranean diet adherence is associated with improved metabolic health, greater medication compliance, and reduced relapse rates (187).

Micronutrients further complement psychopharmacotherapy by supporting neurotransmitter synthesis and cellular function. Deficiencies in B vitamins (B6, B12, folate) and vitamin D are associated with cognitive decline and mood instability, effects that may reinforce the metabolic burden of psychotropic drugs. Supplementation of these vitamins has been shown to enhance neurotransmitter synthesis and may attenuate medication-induced metabolic and neurocognitive side effects (188, 189). Minerals such as magnesium and zinc also improve treatment outcomes and reduce oxidative stress by enhancing neuroplasticity and modulating NMDA and GABA receptors (190, 191). The ketogenic diet, characterized by high fat and low carbohydrate intake leading to ketosis, has been investigated for neuroprotective and mood-stabilizing properties in psychiatric contexts (192). This metabolic shift can modulate neuronal excitability and inflammatory pathways involved in mood disorders. The reduction in glycemic load influences hepatic enzyme activity, affecting the metabolism of cytochrome P450-processed drugs, especially relevant for those on polypharmacy or with hepatic comorbidities (193). The diet’s anti-seizure effect may also complement antiepileptic mood stabilizers, supporting mood stabilization in bipolar disorder (194). However, long-term adherence demands monitoring, as reductions in bone mineral density and changes in lipid profiles and hepatic function may occur (194). While ketogenic diets may be adjunctive in mood stabilization and reduction of cycling in treatment-resistant bipolar disorder, individualized risk–benefit analysis and coordinated monitoring remain essential (192–194). Vegetarian and vegan diets emphasize plant-based foods, fruits, vegetables, legumes, whole grains, nuts, and seeds, with the exclusion of animal products in veganism and the possible inclusion of dairy and eggs in vegetarianism (195). These patterns support an anti-inflammatory milieu and cardiovascular benefit but may contain lower levels of nutrients crucial to psychopharmacotherapy, such as vitamin B12 and marine-sourced omega-3 fatty acids (EPA and DHA) (196–198). Essential amino acids including tryptophan and tyrosine are present but with variable bioavailability, which can affect neurotransmitter synthesis and antidepressant effectiveness (185, 197). Plant-based omega-3 (ALA) converts inefficiently to EPA/DHA, the forms most beneficial for neural health (196, 197). Insufficient EPA/DHA intake may impact membrane fluidity, serotonin receptor activity, and thus mood stabilization and SSRI/SNRI outcomes (185). Algae-derived omega-3 and reliable B12 supplementation represent effective strategies for individuals on psychotropics who prefer plant-based diets (195), and long-term cohorts highlight the importance of proactive nutrient management to prevent deficits that could undermine cognitive and emotional health (198).

Vitamin D is widely distributed in the brain and regulates enzymes necessary for dopamine synthesis. Its deficiency is correlated with mood dysregulation and lower SSRI efficacy (189). Main sources are sunlight, fortified foods, and fatty fish, with supplementation indicated in low sunlight environments (189). B vitamins are essential for neurotransmitter synthesis and homocysteine regulation; deficiency is associated with cognitive decline and reduced efficacy of antidepressants (188, 199, 200). Magnesium modulates NMDA activity and GABAergic tone, benefiting anxiolytic responses and sleep (190, 201). Zinc has antioxidant effects and supports neuroplasticity; its deficiency is associated with diminished antidepressant responses, and supplementation can further reduce depressive symptoms in those taking SSRIs (191, 202, 203). EPA/DHA omega-3 supplementation has demonstrated supportive effects on SSRI/SNRI efficacy, most likely via membrane fluidity and serotonin receptor function, with typical adjunct doses of 1–2 g/day (184, 204). Finally, Targeted probiotic strains modulate the gut–brain axis by influencing neurotransmitter production and attenuating systemic inflammation; in patients receiving SSRIs or anxiolytics, probiotics have been linked to improved mood stability and better gastrointestinal tolerability, helping long-term treatment adherence (205–207).

In summary, integrating tailored nutrition and lifestyle interventions is central to mitigating psychotropic-induced metabolic dysfunction and IR. Clinical guidelines, including those from the American Association of Clinical Endocrinology, recommend balanced, individualized dietary plans, regular physical activity, behavioral self-management, and minimization of tobacco and alcohol use as first-line strategies to address antipsychotic-associated weight gain and cardiometabolic risk (24, 208–210). The Mediterranean diet is best supported for metabolic and neuroprotective benefits, while ketogenic or supplemented plant-based diets may offer individualized advantages. Ensuring adequate micronutrients and targeted probiotics can further enhance neurochemical balance and treatment response. Importantly, social determinants of health, including access to nutritious foods, environments for exercise, and digital health resources, should be addressed to improve adherence and outcomes. Emerging evidence also supports digital health interventions, such as mobile apps and wearables, as valuable tools for overcoming barriers and sustaining lifestyle changes in severe mental illness, though long-term effects need further study (211, 212). Altogether, a multidisciplinary approach combining nutritional, behavioral, and digital strategies, tailored to individual patient needs, offers promise for minimizing metabolic burden and improving physical and mental health outcomes in patients receiving psychotropic medications.

## 10 Can artificial intelligence provide a future solution for patients suffering from psychotropic induced metabolomic disturbance and insulin resistance?

The heterogeneous metabolic consequences of psychotropic medications arise from complex interactions across multiple biological layers, including genomic susceptibility, epigenomic regulation, transcriptomic and metabolomic remodeling, gut microbiome dynamics, and modifiable nutrition-lifestyle factors. While each of these domains has yielded important mechanistic insights, their clinical translation has been limited by fragmentation, high dimensionality, and the difficulty of integrating temporally dynamic, multimodal data into actionable risk stratification or treatment strategies. Traditional statistical approaches are often insufficient to capture nonlinear interactions, cumulative burden, and individual variability across these interconnected systems. Within this context, artificial intelligence (AI) and machine learning (ML) have emerged as promising integrative tools capable of synthesizing multi-omics, clinical, and behavioral data to support early prediction, mechanistic inference, and personalized intervention in psychotropic-induced metabolomic disturbance and IR (12). A key step toward actionable deployment is the prospective protocol by Lee et al. (213), which will enroll 300 patients with severe mental illness (schizophrenia, bipolar disorder, major depressive disorder) and collect baseline and 24-week data spanning demographics, lifestyle, medical history, psychological factors, anthropometrics, and laboratory measures. In Phase 1, ML models are designed to predict psychotropic induced weight gain and metabolic changes over 24 weeks; participants meeting overweight (BMI 23–24.9 kg/m^2^) or obesity (BMI ≥ 25 kg/m^2^) thresholds then enter Phase 2, receive anti-obesity medications for an additional 24 weeks, and undergo repeat assessments to train ML models that predict which anti-obesity drugs are most likely to be effective for each individual. This protocol directly addresses a persistent clinical gap, moving beyond predicting early weight gain to predicting therapeutic reversibility of metabolic toxicity. Parallel AI studies in schizophrenia illustrate how diverse modalities can be leveraged for prediction and personalization: ML using superior temporal cortex functional connectivity in first episode drug naïve schizophrenia achieved 82.5% accuracy in predicting treatment response, with mutual information and correlation based connectivity features contributing strongly (214); ML “brain age” modeling in 2803 schizophrenia cases and 2,598 controls showed schizophrenia brains were 3.55 years older on average, proposing an objective biomarker for disease burden and treatment evaluation (215). At the molecular level, exosome proteomics combined with XGBoost in 343 participants generated a plasma personalized differentiation score (PDS), where higher PDS was significantly associated with greater PANSS improvement after antipsychotic treatment (216). Multi trial ML tools built on first episode schizophrenia trial datasets achieved ~75% accuracy for outcome prediction and identified patients at risk of symptom persistence, poor adherence, and rehospitalization (217, 218). AI has also been used to optimize interventions and implementation: latent class growth modeling in 76 patients undergoing remote social cognitive training (8–12 weeks) identified five response trajectories, including a high response subgroup (29%) whose membership was more accurately predicted by random forest methods; an 8 week computerized cognitive remediation therapy (CCRT) comparison study reported increased resting state activity in DLPFC and ACC with improvements in processing speed and problem solving (219). For rTMS, a sequential prediction workflow integrating structural MRI, clinical and sociodemographic variables, and PRS achieved 94% balanced accuracy and highlighted gray matter density in the default mode network/limbic system plus education related PRS as key predictors (220), while another MRI AI model predicted rTMS response with 85% accuracy (221). Importantly for metabolomic disturbance, graph attention network approaches to psychometabolic interaction modeling identified relationships between psychiatric symptom history and metabolic markers such as triglycerides and LDL C (222), and adherence research indicates that baseline daily functioning predicts adherence to exercise interventions more than symptom severity or fitness (223). For medication management, individualized treatment rules (ITRs) for first episode schizophrenia improved treatment success in training data (51.7% vs. 44.5%), and recommended aripiprazole/amisulpride more often than commonly prescribed agents such as risperidone/sulpiride (224); interpretable AI combining a personalized advantage index with Bayesian rule lists aided paliperidone patient selection (225); and a PANSS score matrix (UPSM) approach delineated five symptom domain subtypes (226, 227). Finally, large scale multimodal AI using EHR + PRS has begun to identify risk and protective factors for antipsychotic metabolic toxicity at scale: in two veteran cohorts (CDW *N* = 869,128; MVP *N* = 137,771 genotyped) using a multimodal BERT architecture, clinically significant weight gain (>7%) was associated with Asian ethnicity, elevated baseline triglycerides, and thiothixene/systemic contraceptives/antipsoriatics, and inversely associated with antimigraine agents, opioid antagonist analgesics, and immune suppressants; in MVP, BMI increase was associated with Hispanic ancestry, first generation antipsychotics, older age, higher T2D PRS, and inversely with BP PRS (227).

Recent advances in AI have also revolutionized prediction and management of drug–nutrient interactions (DNIs) (228), including drug-food (DFIs) (229), drug-supplement (DSIs), and drug-microbiome interactions (DMIs) (230–232). These interactions are especially significant in populations with polypharmacy, such as older adults and individuals with complex psychiatric regimens, where they can lead to micronutrient deficiencies, reduced drug efficacy, and increased adverse effects. AI-driven approaches employing deep neural networks and SMILES-based molecular representations have achieved high accuracy in predicting DFIs, while machine learning methods such as XGBoost, Random Forest, and SVM have characterized enzyme- and transporter-mediated food additive effects (232, 233). Advanced frameworks, including graph-based models like FDMine and DFinder, and natural language processing (NLP)-driven systems, now mine large-scale biomedical literature and clinical notes for DFI and DSI recognition and hypothesis generation. These tools have been particularly useful in delineating clinically significant DSIs, offering novel decision support capabilities for mitigating risk (227, 234, 235). Moreover, evolving evidence underscores the impact of the gut microbiome in drug metabolism. AI models integrating drug chemical structure and microbial genomics can now predict microbial drug transformation, susceptibility to dysbiosis, and resulting metabolic sequelae. Newer pipelines even model probiotic-drug interactions and downstream metabolite production in dynamic GI tract simulations (231, 232, 236, 237).

While methodological advances in artificial intelligence and machine learning have considerably expanded research capabilities in precision psychiatry, it is crucial to critically evaluate their current clinical applicability, particularly in relation to predicting and managing metabolic disturbances associated with psychotropic medication use. Many published models are derived from retrospective datasets, are vulnerable to confounding (e.g., baseline adiposity, illness severity, polypharmacy, and socioeconomic determinants), and can suffer from optimistic performance estimates due to data leakage, small sample sizes, and inconsistent outcome definitions. Generalizability across health systems and ancestries remains a persistent challenge, and model interpretability, calibration, and clinical utility are rarely evaluated in prospective workflows. Recent critical appraisals have argued that a large fraction of existing clinical prediction models is “wrong or useless” without rigorous validation and transparent reporting (13), underscoring the need for caution even if this view is deliberately stringent. Before routine implementation, AI tools in this domain will require robust external validation, harmonized phenotyping, prospective impact assessments (including RCT-embedded evaluations), and careful attention to fairness, privacy, and implementation barriers. In the near term, AI should therefore be viewed as an enabling research and decision-support approach with potential to augment, rather than replace standard metabolic monitoring and clinically grounded risk mitigation strategies.

## 11 Conclusion and future perspectives

As the clinical and mechanistic landscape of psychotropic-induced metabolic dysfunction has evolved, so too has our recognition of the need for system-level, precision approaches in psychiatric care. What was once seen primarily as an inevitable, “side effect” of lifesaving medications is now understood as a modifiable process, driven by the dynamic interplay of host genetics, epigenetic regulation, microbial ecosystems, and environmental exposures, all interwoven through the gut–brain–metabolic axis. Integrative multi-omics technologies, encompassing genomics, epigenomics, transcriptomics, metabolomics and comprehensive microbiome profiling, have elucidated not only the early and heterogeneous onset of metabolic vulnerability, but the molecular, cellular, and ecological networks that make each patient’s trajectory unique.

Crucially, these discoveries are no longer confined to academic discourse. The application of high-dimensional, integrative biomarkers enables the identification of at-risk individuals before the onset of irreversible disease. Omics-driven insights are shaping new paradigms of personalized prevention, therapeutic monitoring, and early intervention, where dietary modulation, pharmacological adjuncts, and targeted microbiome or behavioral therapies can be selected and timed based on individual risk profiles. Dynamic molecular monitoring is strengthening links between psychiatry and primary medical care, while offering renewed hope for bridging the life expectancy gap that has long separated patients with severe mental illness from the general population.

Notwithstanding these advances, the road to real-world impact is still being paved; implementation of science, clinical standardization, digital health integration, and health equity remain active frontiers. The translation of multi-omics discoveries into broadly accessible, iterative, and ethically grounded models of metabolic-psychiatric care will require sustained interdisciplinary collaboration, investment, and attention to the social and environmental determinants of health.

Methodologically, a major limitation of the current literature is the predominance of cross-sectional and case–control study designs, which are highly susceptible to confounding by indication, illness chronicity, baseline adiposity, dietary patterns, smoking, and polypharmacy. Consequently, attributing metabolic alterations specifically to psychotropic exposure versus pre-existing risk remains challenging. Future progress will depend on prospective longitudinal cohorts and randomized controlled trials with embedded mechanistic components, incorporating pre-treatment baselines, repeated integrative multi-omics sampling of host and microbiome biology, and standardized cardiometabolic phenotyping during the early, high-risk phases of treatment. Such designs are essential to establish temporal relationships, strengthen causal inference, and enable clinically actionable prediction models and targeted prevention strategies. Looking ahead, the convergence of big data, AI, digital health tools, and systems biology promise to transform anticipation, even prevention, of metabolic sequelae for all individuals requiring chronic psychotropic therapy. By anchoring practice in mechanistic precision and holistic, patient-centered care, the next chapter of psychiatric medicine offers not only symptom control and improved mental wellbeing, but a tangible path to safeguarding lifelong physical health and quality of life for this vulnerable population.
